# Associations of dietary, sociodemographic, and anthropometric factors with anemia among the Zhuang ethnic adults: a cross-sectional study in Guangxi Zhuang Autonomous Region, China

**DOI:** 10.1186/s12889-023-16697-2

**Published:** 2023-10-06

**Authors:** Zheng Wen, Jianxiong Long, Lulu Zhu, Shun Liu, Xiaoyun Zeng, Dongping Huang, Xiaoqiang Qiu, Li Su

**Affiliations:** 1 Department of Epidemiology and Health Statistics, School of Public Health, Guangxi Medical University, 22 Shuangyong Road, Nanning, 530021 Guangxi China; 2 Guangxi Colleges and Universities Key Laboratory of Prevention and Control of Highly Prevalent Diseases, Guangxi Medical University, Nanning, 530021 Guangxi China; 3 Department of Maternal, Child and Adolescent Health, School of Public Health, Guangxi Medical University, Nanning, Guangxi China; 4 Department of Sanitary Chemistry, School of Public Health, Guangxi Medical University, Nanning, Guangxi China

**Keywords:** Anemia, Hemoglobin, Diet, Guangxi, China, Aging, Red meat, Menopause, Body water percentage

## Abstract

**Background:**

After decades of rapid economic development, anemia remains a significant public health challenge globally. This study aimed to estimate the associations of sociodemographic, dietary, and body composition factors with anemia among the Zhuang in Guangxi Zhuang Autonomous Region, China.

**Methods:**

Our study population from the baseline survey of the Guangxi ethnic minority Cohort Study of Chronic Diseases consisted of 13,465 adults (6,779 women and 6,686 men) aged 24–82 years. A validated interviewer-administered laptop-based questionnaire system was used to collect information on participants’ sociodemographic, lifestyle, and dietary factors. Each participant underwent a physical examination, and hematological indices were measured. Least absolute shrinkage and selection operator (LASSO) regression was used to select the variables, and logistic regression was applied to estimate the associations of independent risk factors with anemia.

**Results:**

The overall prevalences of anemia in men and women were 9.63% (95% CI: 8.94**–**10.36%) and 18.33% (95% CI: 17.42**─**19.28%), respectively. LASSO and logistic regression analyses showed that age was positively associated with anemia for both women and men. For diet in women, red meat consumption for 5–7 days/week (OR = 0.79, 95% CI: 0.65**–**0.98, *p* = 0.0290) and corn/sweet potato consumption for 5–7 days/week (OR = 0.73, 95% CI: 0.55**–**0.96, *p* = 0.0281) were negatively associated with anemia. For men, fruit consumption for 5–7 days/week (OR = 0.75, 95% CI: 0.60–0.94, *p* = 0.0130) and corn/sweet potato consumption for 5–7 days/week (OR = 0.66, 95% CI: 0.46–0.91, *p* = 0.0136) were negatively correlated with anemia. Compared with a normal body water percentage (55–65%), a body water percentage below normal (< 55%) was negatively related to anemia (OR = 0.68, 95% CI: 0.53–0.86, *p* = 0.0014). Conversely, a body water percentage above normal (> 65%) was positively correlated with anemia in men (OR = 1.73, 95% CI: 1.38–2.17, *p* < 0.0001).

**Conclusions:**

Anemia remains a moderate public health problem for premenopausal women and the elderly population in the Guangxi Zhuang minority region. The prevention of anemia at the population level requires multifaceted intervention measures according to sex and age, with a focus on dietary factors and the control of body composition.

**Supplementary Information:**

The online version contains supplementary material available at 10.1186/s12889-023-16697-2.

## Introduction

Hemoglobin (Hb) is a specific protein in red blood cells that delivers oxygen to tissues and returns carbon dioxide to the lungs for elimination from the body [1]. Anemia is a medical syndrome in which the number of red blood cells cannot meet physiological requirements, and it is defined as an Hb concentration lower than normal [2, 3]. The symptoms of anemia originate from impaired tissue oxygen delivery, including fatigue, weakness, cognitive decline, or poor work productivity [4]. In addition, anemia is a common health problem correlated with increased hospitalization and mortality rates among elderly individuals, children, and women [5–7]. Moreover, anemia may be associated with chronic heart failure [8, 9] and infections [10]. Therefore, anemia has a profound impact on increased morbidity and mortality.

Globally, the prevalence of anemia in 2010 was 32.9% [11]. Specifically, anemia affects 1.93 billion people throughout the world (approximately one-third of the world’s population), and developing countries are responsible for more than 89% of this burden [12]. In China, anemia affects 31.0% [13] of the Chinese population and remains the most significant nutritional disorder. In most countries, anemia epidemiology varies based on geographic regions, ethnic groups, and socioeconomic factors [14]. China is a multiethnic country composed of Han nationality and 55 ethnic minorities. Most ethnic minorities live in remote areas in China with different geographies, environments, and climates or with a single dietary pattern. However, with the development of the economy, the dietary habits and lifestyles of ethnic minorities have gradually changed. Therefore, it is necessary to clarify the prevalence of anemia and to explore the factors related to anemia at the population level, especially in areas where ethnic minorities gather.

Th Guangxi Zhuang Autonomous Region (Guangxi) is located in mountainous terrain far south of China and is famous for karst landforms. Guangxi has abundant rainfall and sufficient sunlight in the subtropical monsoon climate region. Moreover, Guangxi is a less-developed border province with unique cultural diversity [15]. The Zhuang population is the largest minority nationality in China. A total of 31.36% (15.72 million) of the population of Guangxi belongs to the Zhuang [16], which accounts for more than 95% of the Zhuang population in China [17]. Most Zhuang minorities live in economically underdeveloped rural areas, especially elderly individuals. Similar to traditional Chinese southern dietary patterns [18], the dietary patterns of Guangxi Zhuang are characterized by high consumption of rice and fresh leafy vegetables; moderate consumption of red meat, poultry, fish, or seafood; and low milk and dessert consumption. Previous studies have demonstrated a high thalassemia incidence in Zhuang populations in Guangxi [17, 19, 20]. However, population-based studies on the prevalence of anemia in minority areas of Guangxi are lacking. In addition, the association of diet, physical activity, and anthropometric variables with anemia is still debatable, especially due to the fact there are few related studies from ethnic minority areas in Guangxi.

With the development of information technology, more variables are available for exploring disease risk factors. However, due to the multicollinearity between predictor variables, the correct identification of the factors that play a vital role in disease development in a set of candidate variables becomes challenging. Furthermore, multicollinearity can cause the coefficient estimates of the model to be unreliable and exhibit high variance [21]. In addition, conventional regression models, including a large number of covariates, are subject to overfitting [22]. Currently, penalized or regularization regression techniques can optimally address these problems. Moreover, least absolute shrinkage and selection operator (LASSO) regression, which is a shrinkage and variable selection method for regression models, is an attractive option because it addresses both problems [23]. LASSO regression automatically selects significant variables and shrinks the coefficients of unimportant predictors to zero [24].

Altogether, the identification of epidemiological characterization regarding the prevalence and distribution of the type of anemia is critical for appropriate prevention strategies in regions with a high prevalence of anemia. Therefore, based on baseline data of the Guangxi Ethnic Minority Cohort Study of Chronic Diseases (GEMCSCD), we aimed to estimate the prevalence and sex-specific risk factors for anemia in ethnic minority areas in Guangxi, southern China. Our study will provide a reference for formulating policies to prevent anemia in countries and regions with similar geographical characteristics or economic levels.

## Methods

### Sites, sample size, and study population

The study was conducted in three regions in Guangxi, namely Nanning, Liuzhou, and Baise, from May 2018 to November 2019. Specifically, we recruited rural participants from 57 villages in five townships in the Liangqing District and six villages in Mashan County in Nanning. In addition, we recruited urban participants from the Physical Examination Department of Liuzhou People’s Hospital and Liuzhou Worker’s Hospital, as well as from Guangxi Liuzhou Iron and Steel Group in Liuzhou and Guangxi Ping Aluminum Group in Pingguo County, Baise. We selected these survey sites based on population stability and economic disparities. These regions consisted both rural and urban settings at different levels of development and contain a high proportion of the Zhuang population.

The sample size for this study was estimated according to the formula for calculating the sample size in cross-sectional studies [25, 26], which is $n=\frac{{z}_{\alpha }^{2}\times \text{p}(1-\text{p})}{{d}^{2}}$. In this formula, n represents the required sample size, p represents the expected proportion in the population based on previous studies, ${z}_{\alpha }$ represents the critical value of the standard normal distribution at a 95% confidence interval (CI) (1.96), and d represents the desired margin of error or precision (set at 10% of the expected proportion, d = 0.1p). A previous study reported the prevalence of anemia among Chinese rural residents was 7.2% (95% CI: 6.8–7.7) for men and 12.4% (95% CI: 11.9–12.9) for women [27]. Therefore, the expected prevalence of anemia was estimated as 6.8% (0.068) for men and 11.9% (0.119) for women. Based on these expected prevalence values, the sample size required for men was 5,265 individuals, while for women, it was 2,844. Considering an additional 20% for potentially invalid questionnaires, the sample sizes at least needed were 6,318 for men and 3,412 for women.

Potential participants were recruited through a range of strategies, including door-to-door delivered invitation letters (with study information leaflets) by local community leaders or health workers, posters posted on-site or community centers, and face-to-face invitations on-site. The recruitment criteria were as follows: (1) local permanent residents; (2) age greater than 18 years; (3) without severe physical disability or mental illness; and (4) if female, the subject must not be pregnant or breastfeeding at the time of recruitment. No exclusion criteria were applied, except for those who did not complete medical examinations or blood sample collection. In total, 14,073 adults (7,041 women and 7,032 men) were recruited. Of these, 564 (4.01%) participants who did not finish the medical examination or blood sample collection and 44 (0.33%) participants missing the identification ID were excluded from the analysis. Participants were enrolled in GEMCSCD, which was designed to elucidate the incidence trend and causes of chronic diseases [28]. The Guangxi ethnic minority area is one of the investigation points of the South China Cohort (SCC) [29]. This study utilized baseline data from GEMCSCD and thoroughly used Hb-related data to clarify the epidemiological characterization of anemia. The study was approved by the Ethics Committee of Guangxi Medical University (No. 20,170,201–1), and all participants signed informed consent before being recruited into the study.

### Data collection and measurement

Data on participants’ sociodemographic characteristics, including age, sex, ethnicity, marital status, highest educational attainment, total household income per year, residence (urban signifies city, and rural represents town or countryside), and the number of families living together (living alone or living with family members), were collected. The participants’ self-reported physician diagnosis history of chronic diseases, including hypertension, diabetes, coronary heart disease (CHD), stroke, and cancer at any site, were collected. Participants who had at least one parent, sibling, or child with any chronic diseases mentioned above were defined as having a family history of chronic disease positivity [30–32]. Moreover, dietary intake over the past year was acquired by the food frequency questionnaire (FFQ) of traditional food [33, 34]. Participants’ physical activity was divided into vigorous-intensity activity, moderate-intensity activity, walking, and sedentary level, which was evaluated by recalling the past 7 days [35, 36]. Occupational activities and recreational activities were included in the calculation of exercise exposure. The vigorous-intensity activity was defined as participants feeling a markedly increased heart rate and shortness of breath, as well as activities lasting for more than 10 min, such as running or playing basketball. The moderate-intensity activity was defined as participants breathing slightly faster than usual, such as hoeing, cycling at a typical speed, and ballroom dancing (but not including walking). Walking includes work-related activities, daily living activities, and pleasure walking lasting more than 10 min on a daily basis [37, 38]. The sedentary level was measured as time spent sitting, and it was calculated based on the self-reported number of hours per day. Moreover, smoking was defined as at least 1 cigarette per day or ≥ 7 cigarettes per week lasting six months or longer [39, 40]. Two categories were defined for smoking: never smoking and current or former smoking statuses. Alcohol consumption was defined as drinking alcoholic beverages in the past 12 months [41]. Alcohol consumption was divided into three categories: never drinking, drinking < 1 time per week, and drinking ≥ 1 time per week [42]. Additionally, the participants’ information on tea consumption was obtained in the past 12 months and divided into three categories: never drinking, drinking a few times a year, and drinking ≥ 1 time per week [43]. Participants’ sleep quality was assessed by using the Pittsburgh Sleep Quality Index scale (PSQI), in which a global score > 5 was defined as poor sleep quality [44]. For the female participants, we also collected menstrual and reproductive history data.

We used a website electronic questionnaire system for on-site questionnaire surveys via face-to-face interviews to ensure the integrity and traceability of the data. The system can record the questionnaire, upload the questionnaire recording, monitor the questionnaire duration, and automatically conduct a logical review. All of the investigators were trained to administer questionnaires by using the electronic questionnaire system. Shanghai Avntech Biotechnology Co., Ltd. technically supported the research management platform.

### Blood samples, Hb measurement, and criteria for anemia

After an overnight fast for at least 8 h, 5 mL of peripheral whole venous blood samples were collected in ethylene diamine tetra-acetic acid (EDTA) tubes for routine blood testing. Hb, mean corpuscular volume (MCV), and mean corpuscular hemoglobin concentration (MCHC) were measured by using an the automatic modular blood and body fluid analyzer (Sysmex NX 10 [B4], Japan). Hb cutoff concentrations for the diagnosis of anemia were < 13.0 g/dL for men and < 12.0 g/dL for non-pregnant women, according to the WHO recommendations [45]. Anemia type was further categorized according to MCV and MCHC measurements. Microcytosis, normocytosis, and macrocytosis were defined as MCV < 80 fL, MCV = 80–100 fL, and MCV > 100 fL, respectively. Hypochromia, normochromia, and hyperchromia were defined as an MCHC < 32 g/dL, MCHC = 32–36 g/dL, and MCHC > 36 g/dL, respectively [46].

### Anthropometric variables

Each participant underwent a physical examination by trained medical staff. Participants were required to keep calm for at least 10 min before blood pressure measurements, and blood pressure was measured two times and averaged. Body fat rate, body water percentage, visceral fat level, lean muscle mass, and bone mass were assessed by using a body composition analyzer (TANITA BC-601, Japan). Lung carbon monoxide (CO) concentration was an index for biochemically established smoking status, and it was assessed by using a noninvasive monitor (Smokerlyzer LAB658, Bedfont, England). Furthermore, body mass index (BMI) was calculated, and waist circumference and hip circumference were measured. Abdominal obesity was defined as waist circumference (WC) > 90 cm in men and WC > 80 cm in women [47]. Moreover, the waist-hip rate (WHR) was calculated by dividing the waist by the hip circumference.

### Statistical analysis

Given the sex differences in the Hb concentrations and physiological variations, we conducted our analysis separately by sex. The continuous variables were verified for normality by using Kolmogorov–Smirnov tests. Continuous variables that did not meet normality were described by using the median and interquartile range (IQR), and the Wilcox test was used to detect statistical significance. Categorical variables were expressed as frequency (n) and proportions (%) and tested via chi-square. Univariate logistic regression was used to explore the association of sex, ethnicity, and residence with anemia.

LASSO regression was used for the variable selection. By imposing a penalty λ on the absolute value of the coefficient of ${\beta }_{j}$ on the coefficient estimate (i.e., L1 regularization: $\lambda \sum _{j=1}^{p}\mid {\beta }_{j}\mid$, where p is the number of variables, and ${\beta }_{j}$ is the effect of variable j), LASSO can force the coefficients of less contributive variables to zero (discarded variables with negligible effects) and keep the most significant variables in the model [48, 49]. We applied 10-fold cross-validation during the LASSO regression. The binomial deviance was calculated for a series of λ values, and the λ of the minimum binomial deviance plus one standard error (SE) was selected as the optimal penalization parameter. ORs (95% CIs) were calculated by using logistic regression to estimate the association of independent risk factors with anemia after variable selection performed by LASSO. All of the analyses were conducted in R (version 4.0.2). The glmnet package was used for LASSO regression. Furthermore, the statistical significance level that was used in this study was a two-tailed α of 0.05.

## Results

### Study population

Finally, we included 13,465 participants in this study, of which 6,779 were women (50.35%) and 6,686 were men (49.65%). The median (IQR) age of this population was 53 (45–63) years (range: 24–82 years). Of the participants, 12,419 (92.23%) were Zhuang, and 1,046 (7.77%) were from other ethnicities. A total of 10,461 (77.69%) participants lived in rural areas, and 3,004 (22.31%) participants lived in urban areas (Table 1).

**Table 1:** Prevalence of anemia according to categories of sex, ethnicity, and residence

Characteristic	Total, n (%)	Anemia, n (%)	Prevalence of anemia, % (95% CI)	OR (95% CI)	*p*^1^	Hemoglobin (Median, IQR), g/dL	*p*^2^
Sex					< 0.0001		< 0.0001
Men	6,686 (49.65)	644 (34.13)	9.63 (8.93–10.36)	1.00 (ref.)		15.00 (14.00–15.80)	
Women	6,779 (50.35)	1,243 (65.87)	18.34 (17.42–19.28)	2.11 (1.90–2.33)		13.10 (12.30–13.90)	
Ethnicity					0.0139		< 0.0001
Others	1,046 (7.77)	120 (6.36)	11.47 (9.60–13.56)	1.00 (ref.)		14.40 (13.30–15.30)	
Zhuang	12,419 (92.23)	1,767 (93.64)	14.23 (13.62–14.85)	1.28 (1.06–1.57)		13.90 (12.80–15.00)	
Residence					< 0.0001		< 0.0001
Urban	3,004 (22.31)	332 (17.59)	11.05 (9.95–12.23)	1.00 (ref.)		14.50 (13.40–15.50)	
Rural	10,461 (77.69)	1,555 (82.41)	14.86 (14.19–15.56)	1.41 (1.24–1.60)		13.80 (12.80–14.90)	

^1^Univariate logistic regression was used

^2^Wilcox test was used

The majority of the participants had a primary school education (*n* = 4,859, 36.09%), followed by junior high school (*n* = 3,964, 29.44%) and high school education (*n* = 2,016, 14.97%). A total of 12.54% (*n* = 1,688) did not complete primary education, and 6.97% (*n* = 938) had a college education or higher. More than half (*n* = 6,737) of the study population worked in agriculture. Additionally, most (*n* = 11,969, 88.89%) of the participants were married, and 11.11% (*n* = 1,496) were unmarried, widowed, or divorced. Regarding tobacco consumption, 0.18% (*n* = 12) of the female participants and 52.33% (*n* = 3,499) of the male participants were current or former smokers. In terms of alcohol consumption, among the male participants, 31.57% (*n* = 2,111) were nondrinkers, 42.39% (*n* = 2,834) drank less than once a week, and 26.04% (*n* = 1,741) consumed at least once a week. In contrast, 90.35% (*n* = 6,125) of female participants were nondrinkers, 9.04% (*n* = 613) drank less than once a week, and 0.60% (*n* = 41) drank at least once a week. Among all of the participants, 33.86% (*n* = 4,559) were overweight, and 9.82% (*n* = 1,322) were obese, and 5.52% (*n* = 743) were underweight. According to the Pittsburgh Sleep Quality Index, 32.26% (*n* = 4,344) of the participants had poor sleep quality. Furthermore, self-reported rates of physician-diagnosed hypertension, diabetes, coronary heart disease, and stroke were 14.76% (*n* = 1,988), 3.21% (*n* = 432), 1.19% (*n* = 160), and 0.88% (*n* = 119), respectively.

### Prevalence and type of anemia

**Fig. 1:**
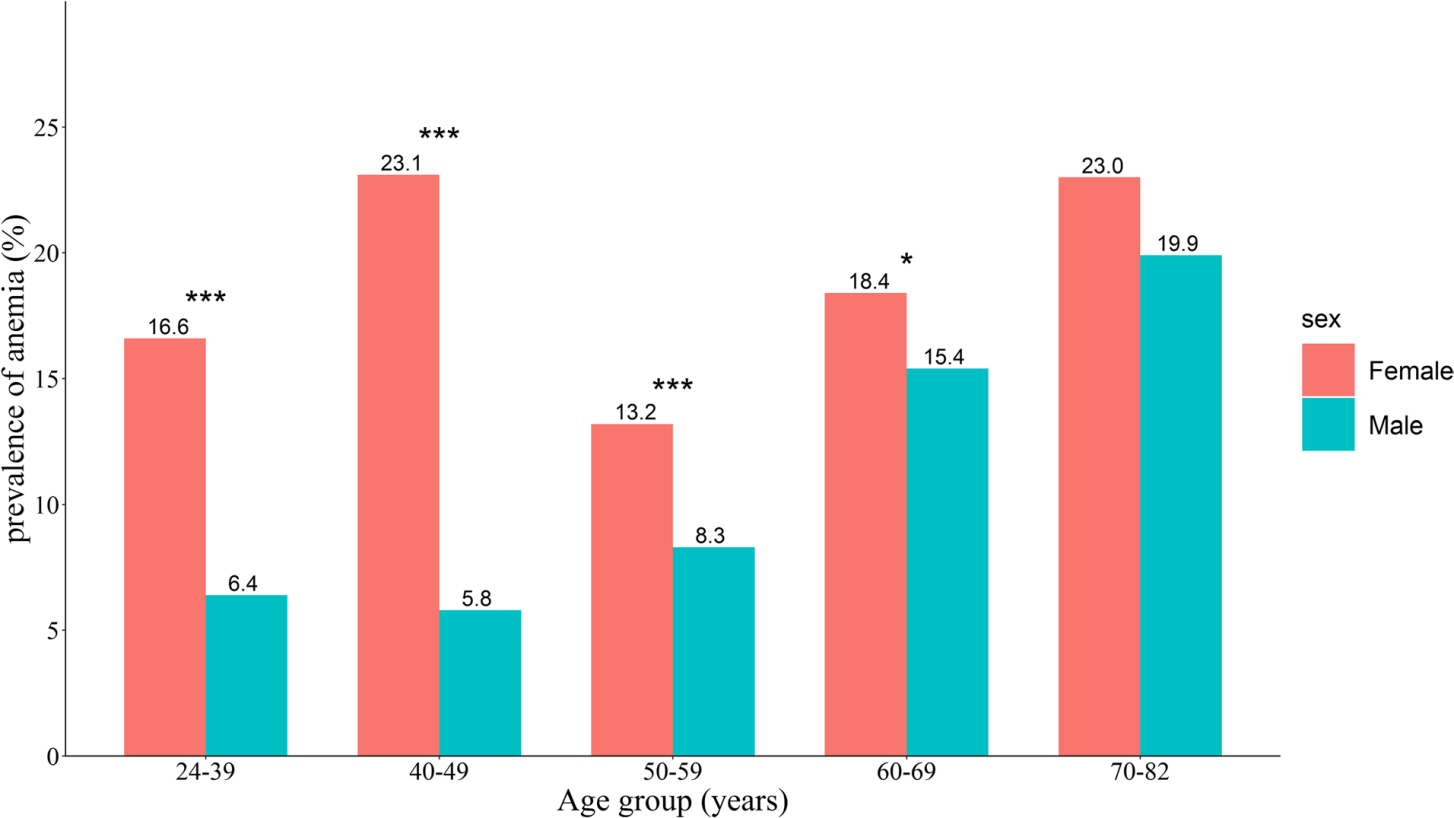
The prevalence of anemia in both men and women by age group. *p < 0.05, ***p < 0.0001

**Fig. 2:**
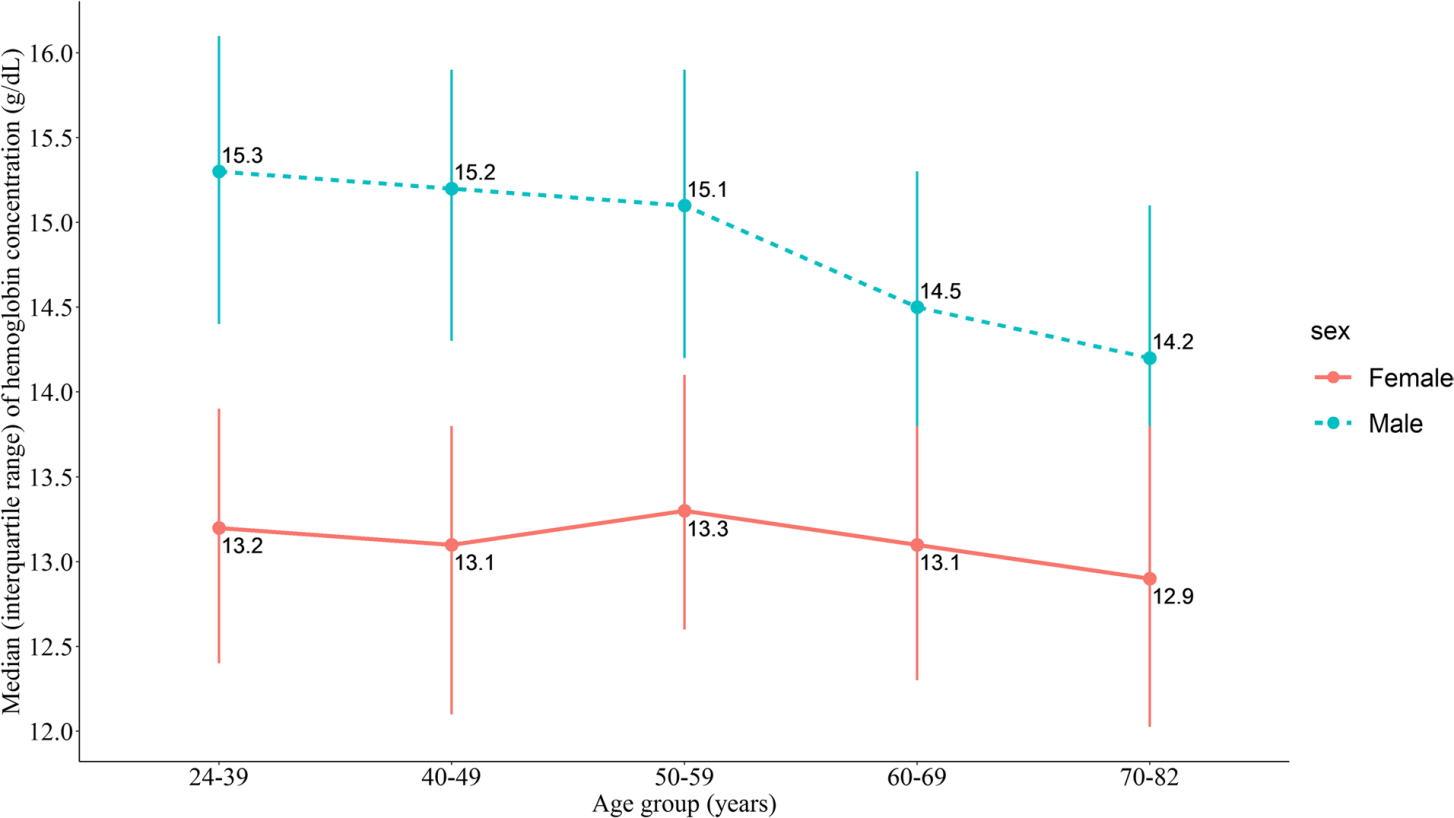
The median and interquartile range Hb concentration in age groups of men and women

The median (IQR) of Hb concentrations were 15.00 (14.00–15.80) g/dL for men and 13.10 (12.30–13.90) g/dL for women. The prevalence of anemia in men and women was 9.63% (95% CI: 8.94–10.36%) and 18.33% (95% CI: 17.42–19.28%), respectively. Women had lower Hb concentrations (*p* < 0.0001) and a higher prevalence of anemia (*p* < 0.0001), and they were more likely to suffer from anemia than men (OR = 2.11, 95% CI: 1.90–2.33, *p* < 0.0001). As shown in Fig. 1, the prevalence of anemia in women in each age group was higher than that in men (*p* < 0.05), except for the 70–82 age group. Notably, we observed that with increasing age, the median of Hb concentration decreased, and the prevalence of anemia increased in men (*p* for trend < 0.001, Figs. 1 and 2). As shown in Table 1, the Zhuang population had lower Hb concentrations than the other ethnicities, and the overall Zhuang population was more prone to anemia than the other ethnicities (OR = 1.28, 95% CI: 1.06–1.57, *p* = 0.0139). Participants living in urban areas had higher Hb concentrations than those living in rural areas (*p* < 0.0001), and the median (IQR) Hb concentrations of urban and rural residents were 14.50 (13.40–15.50) g/dL and 13.8 (12.80–14.90) g/dL, respectively. Furthermore, rural residents were at a higher risk than urban residents (OR = 1.41, 95% CI: 1.24–1.60, *p* < 0.0001).

Table 2 shows the proportion of anemia types according to MCV and MCHC measurements. The highest proportion was hypochromic microcytic anemia, followed by hypochromic normocytic and normochromic normocytic anemia in all of the age groups for both men and women.

**Table 2:** The proportion of anemia types according to the mean corpuscular volume and mean corpuscular hemoglobin concentration of all anemia population

Characteristic	Anemia types, n (%)				*p*^*1*^
	Hypochromic Microcytic	Hypochromic Normocytic	Normochromic Normocytic	Others^2^	
**Total**	1,179 (62.48%)	445 (23.58%)	181 (9.59%)	82 (4.35%)	
**Sex**					< 0.0001
Female	777 (62.51%)	318 (25.58%)	111 (8.93%)	37 (2.98%)	
Male	402 (62.42%)	127 (19.72%)	70 (10.87%)	45 (6.99%)	
**Age**					< 0.0001
24–39	95 (69.34%)	24 (17.52%)	11 (8.03%)	7 (5.11%)	
40–49	344 (66.41%)	104 (20.08%)	49 (9.46%)	21 (4.05%)	
50–59	281 (67.87%)	84 (20.29%)	33 (7.97%)	16 (3.86%)	
60–69	354 (60.31%)	147 (25.04%)	59 (10.05%)	27 (4.60%)	
70–82	105 (45.45%)	86 (37.23%)	29 (12.55%)	11 (4.76%)	
**Female**					0.0146
24–39	60 (66.67%)	20 (22.22%)	9 (10.00%)	1 (1.11%)	
40–49	243 (62.47%)	95 (24.42%)	39 (10.03%)	12 (3.08%)	
50–59	185 (70.08%)	58 (21.97%)	16 (6.06%)	5 (1.89%)	
60–69	222 (61.84%)	92 (25.63%)	33 (9.19%)	12 (3.34%)	
70–82	67 (47.52%)	53 (37.59%)	14 (9.93%)	7 (4.96%)	
**Male**					< 0.0001
24–39	35 (71.47%)	4 (8.51%)	2 (4.26%)	6 (12.77%)	
40–49	101 (78.29%)	9 (6.98%)	10 (7.75%)	9 (6.98%)	
50–59	96 (64.00%)	26 (17.33%)	17 (11.33%)	11 (7.33%)	
60–69	132 (57.89%)	55 (24.12%)	26 (11.40%)	15 (6.58%)	
70–82	38 (42.22%)	33 (36.67%)	15 (16.67%)	4 (4.44%)	

^1^The chi-squared test was used

^2^Others include: Normochromic Microcytic: 37 (1.96%); Hypochromic Macrocytosic: 22 (1.17%); Normochromic Macrocytosic 22 (1.17%); Hyperchromic Macrocytosic: 1 (0.05%)

### Association between anemia and relative variables

Based on sociodemographic, lifestyle, dietary, medical examination, chronic disease state, physical activity, and sleep factors, sex-separated anemia status is shown in Supplement Tables 1-5. Supplement Table 6 shows that menopausal status was related to anemia in females (*p* < 0.001). We did not observe that the age at the first menarche, the number of pregnancies, and the number of live births were statistically significant between women with anemia and without anemia (*p* > 0.05).

**Fig. 3:**
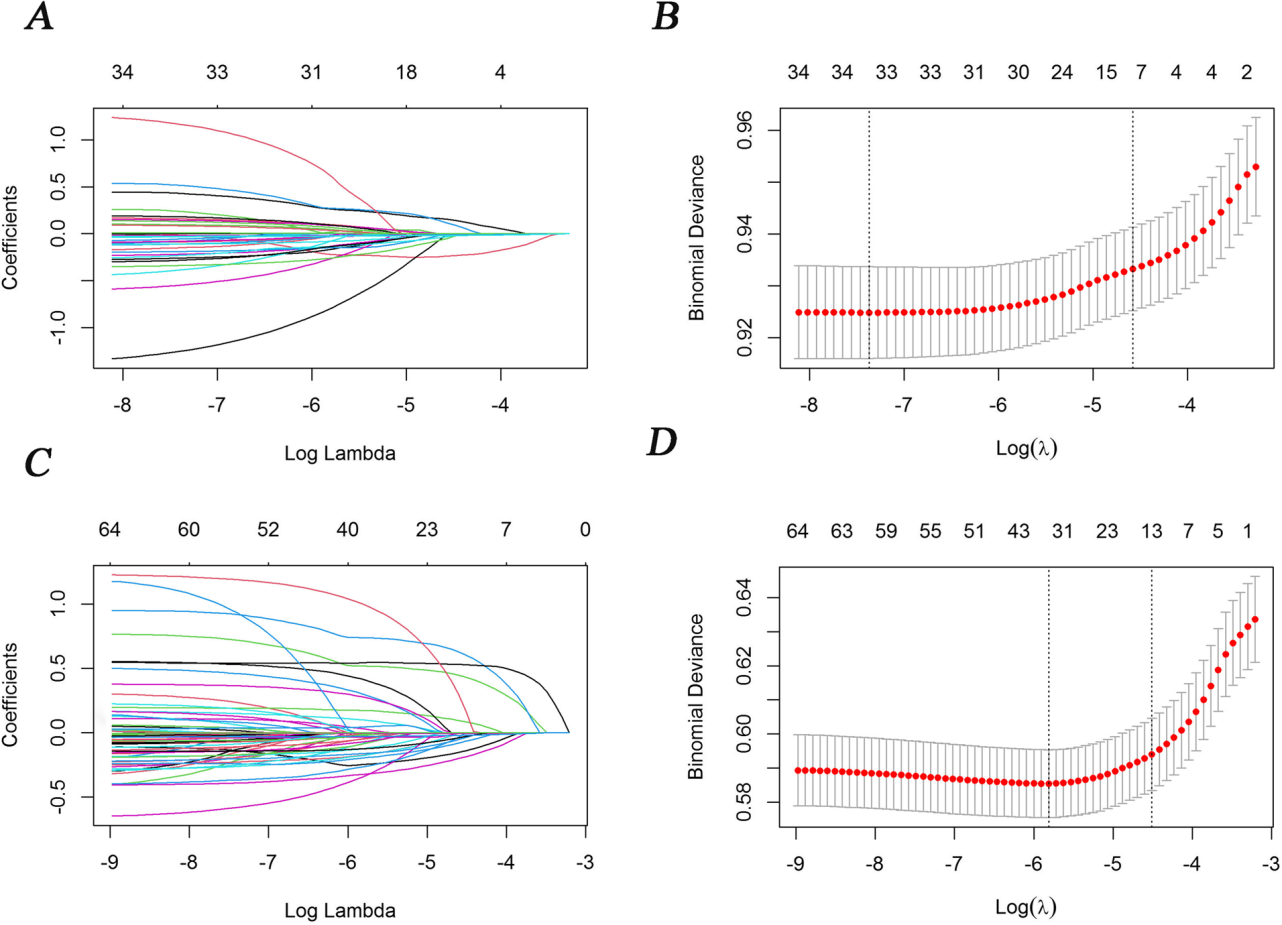
LASSO regression model coefficients over different penalty parameters for women and men. **A** and **C** represent the changing trend of coefficient variables and the log λ for women and men, respectively. Each line represents one variable. **B** and **D** represent the LASSO regression 10-fold cross-validation process for women and men, respectively. The red dots represent mean binomial deviance by using 10-fold cross-validation. The left vertical dashed line indicates the minimum binomial deviance λ value. The right vertical dashed line indicates the minimum plus one SE λ value, which was the optimal lambda via 10-fold cross-validation by using binomial deviance

We performed LASSO regression to identify the possible risk factors for women. A total of 20 variables were enrolled in LASSO, and 8 variables were related to anemia in women. Table 3 shows the variables that were related to anemia in LASSO and the logistic regression model for women. After performing LASSO for variable selection, the logistic regression showed that women aged 40–49, 60–69, and 70–82 years were at a higher risk than women aged 24–39 years (OR = 1.67, 95% CI: 1.29**–**2.17, *p* = 0.0001; OR = 1.64, 95% CI: 1.19**–**2.27, *p* = 0.0025; OR = 2.37, 95% CI: 1.66–3.41, *p* < 0.0001, respectively). Furthermore, menopause was negatively associated with anemia in women (OR = 0.73, 95% CI: 0.59–0.89, *p* = 0.0019). Regarding diet in women, red meat consumption for 5–7 days/week (OR = 0.79, 95% CI: 0.65–0.98, *p* = 0.0290) and corn, sweet potato, and other miscellaneous grains consumption for 5–7 days/week (OR = 0.73, 95% CI: 0.55–0.96, *p* = 0.0281) were inversely associated with anemia, compared with consumption ≤ 3 times/month. In contrast, rice consumption ≤ 3 times/month (OR = 0.24, 95% CI: 0.04–0.78, *p* = 0.0487) was inversely associated with anemia compared with consumption for 5–7 days/week. In addition, we obsered that diastolic blood pressure (OR = 0.98, 95% CI: 0.97–0.99, *p* < 0.0001) and body fat percentage (OR = 0.97, 95% CI: 0.96–0.98, *p* < 0.0001) were negatively correlated with anemia in women. In addition, the visceral fat level was not associated with anemia in women. Figure 3A shows the coefficient trend when imposing penalty λ, and B shows LASSO 10-fold cross-validation for women.

**Table 3:** The coefficients of variables in LASSO and logistic regression models with the risk of anemia for women

Characteristic	LASSO	Logistic		
	β	β	OR (95% CI)	*p*
Age				
24–39	ref.	ref.	ref.	
40–49	0.1571	0.5110	1.67 (1.29–2.17)	0.0001
50–59	−0.2397	0.0901	1.09 (0.81–1.49)	0.5572
60–69	0	0.4968	1.64 (1.19–2.27)	0.0025
70–82	0.1190	0.8644	2.37 (1.66–3.41)	< 0.0001
Menopause				
No	ref.	ref.	ref.	
Yes	−0.0002	−0.3190	0.73 (0.59–0.89)	0.0019
Visceral fat level				
Normal (level:1–4)	ref.	ref.	ref.	
Mild (level:5–9)	0	0.1189	1.13 (0.93–1.37)	0.2304
Moderate (level:10–14)	−0.0025	−0.1650	0.85 (0.57–1.25)	0.4123
High (level: ≥ 15)	0	−0.4899	0.61 (0.14–1.82)	0.4349
Rice				
5–7 days/week	ref.	ref.	ref.	
1–4 days/week	0	−0.6038	0.55 (0.19–1.29)	0.2131
≤ 3 times/month	−0.0107	−1.4415	0.24 (0.04–0.78)	0.0487
Corn/sweet potato				
≤ 3 times/month	ref.	ref.	ref.	
1–4 days/week	0	−0.0513	0.95 (0.82–1.10)	0.5035
5–7 days/week	−0.0529	−0.3135	0.73 (0.55–0.96)	0.0281
Red meat				
≤ 3 times/month	ref.	ref.	ref.	
1–4 days/week	0	−0.0768	0.93 (0.75–1.15)	0.4885
5–7 days/week	−0.0331	−0.2323	0.79 (0.65–0.98)	0.0290
DBP	−0.0118	−0.0173	0.98 (0.97–0.99)	< 0.0001
Body fat rate (%)	−0.0195	−0.0298	0.97 (0.96–0.98)	< 0.0001

*Abbreviations*: *LASSO* least absolute shrinkage and selection operator, *Ref* reference, *DBP* diastolic blood pressure, *CI* confidence interval, *OR *odds ratio

The variables associated with anemia in men in the LASSO and logistic regression analyses are shown in Table 4. Compared with the age range of 24–39 years, those of 60–69 (OR = 2.33, 95% CI: 1.66–3.34, *p* < 0.0001) and 70–82 (OR = 3.08, 95% CI: 2.07–4.63, *p* < 0.0001) years were positively associated with anemia, whereas the age ranges of 40–49 and 50–59 years were not correlated with anemia. Among dietary factors, the consumption of corn, sweet potato, and other miscellaneous grains (OR = 0.66, 95% CI: 0.46–0.91, *p* = 0.0136) and fruit for 5–7 days/week (OR = 0.75, 95% CI: 0.60–0.94, *p* = 0.0130) was negatively correlated with anemia in men compared with consumption ≤ 3 times/month. Notably, compared with a normal body water percentage (55–65%), a body water percentage below normal (< 55%) was negatively related to anemia (OR = 0.68, 95% CI: 0.53–0.86, *p* = 0.0014). Conversely, a body water percentage above normal (> 65%) was positively correlated with anemia in men (OR = 1.73, 95% CI: 1.38–2.17, *p* < 0.0001). Moreover, DBP (OR = 0.97, 95% CI: 0.96–0.97, *p* < 0.0001) was negatively correlated with anemia in men. Cancer was positively correlated with anemia (OR = 3.62, 95% CI: 1.28–9.48, *p* = 0.0106). The variable selection process in men based on LASSO regression coefficients is shown in Fig. 3C and D.

**Table 4:** The coefficients of variables in LASSO and multivariate logistic regression with the risk of anemia for men

Characteristic	LASSO	Logistic		
	β	β	OR (95% CI)	*p*
Age				
24–39	ref.	ref.	ref.	
40–49	−0.1420	−0.0716	0.93 (0.66–1.33)	0.6890
50–59	0	0.3337	1.40 (0.99–2.00)	0.0633
60–69	0.4326	0.8479	2.33 (1.66–3.34)	< 0.0001
70–82	0.6048	1.1254	3.08 (2.07–4.63)	< 0.0001
BMI (kg/m2)				
Normal (18.5–23.9)	ref.	ref.	ref.	
Underweight (< 18.5)	0.0834	0.1919	1.21 (0.87–1.67)	0.2459
Overweight (24–27.9)	−0.0676	−0.2094	0.81 (0.64–1.02)	0.0781
Obesity (≥ 28)	0	0.1433	1.15 (0.76–1.72)	0.4895
Body water percentage (%)				
Normal (male: 55–65)	ref.	ref.	ref.	ref.
Below normal (male: < 55)	−0.1867	−0.3926	0.68 (0.53–0.86)	0.0014
Above normal (male: > 65)	0.5295	0.5497	1.73 (1.38–2.17)	< 0.0001
Corn/sweet potato				
≤ 3 times/month	ref.	ref.	ref.	
1–4 days/week	0	0.0417	1.04 (0.84–1.28)	0.6989
5–7 days/week	−0.0014	−0.4212	0.66 (0.46–0.91)	0.0136
Fresh fruit				
≤ 3 times/month	ref.	ref.	ref.	
1–4 days/week	0	−0.0767	0.93 (0.74–1.15)	0.4957
5–7 days/week	−0.0385	−0.2833	0.75 (0.60–0.94)	0.0130
Cancer				
No	ref.	ref.	ref.	
Yes	0.1687	1.2864	3.62 (1.28–9.48)	0.0106
DBP	−0.0202	−0.0345	0.97 (0.96–0.97)	< 0.0001
Lean muscle mass (kg)	−0.0070	−0.0052	0.99 (0.97–1.02)	0.6096
Bone mass	−0.0300	−0.1583	0.85 (0.60–1.12)	0.2999

*Abbreviations*: *LASSO* least absolute shrinkage and selection operator, *Ref* reference, *BMI* body mass index, *DBP* diastolic blood pressure

## Discussion

This study provides the prevalence of anemia and associated factors in the Guangxi Zhuang minority region of South China. Our research showed that the overall prevalence of anemia in the Guangxi Zhuang minority region was 14.01% (95% CI: 13.43–14.61%), which was lower than the global anemia prevalence (32.9%) but higher than the Chinese rural anemia prevalence (9.7%) in 2010 [27]. In 2014, a cross-sectional study of 10 ethnic minorities in China showed that the prevalence of anemia in Zhuang women aged 20‒49 years was 26.6% [50]. In our study, the prevalence of anemia in Zhuang women aged 24‒49 years was 20.94% (416/1,987), which was statistically lower than that of previous studies (χ^2^ = 19.654, *p* < 0.0001). This is consistent with the decreased global prevalence of anemia reported from 1990 to 2019 [51]. The decreasing prevalence of anemia may result from the continuous improvement of living standards of people in the Guangxi Zhuang minority region and better access to health care services. However, the prevalence of anemia in the Guangxi Zhuang minority region is still higher than that in developed countries. For instance, a recently published study that included 85,992 participants in the United States from 1999 to 2020 showed that the prevalence of anemia in America was 7.30% (95% CI: 7.40–8.19%) for women and 3.69% (95% CI: 3.42–3.97%) for men [52]. Similarly, compared with developing countries in South America, the prevalence of anemia among women in Guangxi is higher than that in developing countries such as Chile (5.1%) and Colombia (7.6%) [53]. Moreover, compared with the Asian region, a survey involving 62,825 Korean individuals from 2007 to 2016 demonstrated that the prevalence of anemia was 7.3% (95% CI: 7.1–7.5%) in South Korea [54]. One of the critical reasons for this result may be that Guangxi is the main concentration of the Zhuang population. Studies have shown that 19.10–24.51% of the Guangxi population carries a potentially pathological hemoglobinopathy gene [17, 19, 20], which may contribute to the high prevalence of anemia in Guangxi. However, it is worth noting that Guangxi was an area with a high rate of malaria transmission in the past [55]. Specifically, people in Guangxi have been combatting malaria for a long period of time. Therefore, the high prevalence of inherited Hb disorders (such as thalassemia) in the Guangxi Zhuang minority region may be due to the mechanism of resistance to malaria resulting from gene-environment interaction for generations [56].

With more in-depth analyses in our study, we found that the most common anemia type was hypochromic microcytic, followed by hypochromic normocytic and normochromic normocytic anemias. These results indicate that the leading causes of anemia in the Guangxi Zhuang area are iron deficiency, vitamin B6 deficiency, thalassemia, chronic blood loss, and aplastic anemia. Thus, there is an urgent need to focus on modifiable risk factors (such as diet and lifestyle) that influence the development of anemia, in order to implement effective prevention measures in the Guangxi Zhuang minority region.

We used LASSO regressions separately for men and women in the analysis to assess the most contributive variables related to anemia. To the authors’ knowledge, this study is the first to explore the association of factors such as diet, sociodemographic characteristics, lifestyle, and physical examination with anemia by using a cross-sectional study combined with LASSO regression for variable selection. Our data demonstrated that the risk of anemia is higher in the 40–49, 60–69, and 70–82 age groups than in the 24–39 age group; however, this was not observed in women aged 50–59. Furthermore, both LASSO and logistic regression analyses confirmed that menopause has a negative correlation with anemia. Women around the age of 50 years are in menopause and typically experience a disappearance of iron-deficiency anemia caused by menstrual blood loss, which is in conjunction with findings from a previous study conducted on women in the reproductive age group of 15–49 years [57]. Furthermore, given the low absorption rate of nutrition and decreased hematopoietic function observed later in life, the older men and women have a higher risk of anemia. The previous research performed on older female adults aged at least 60 years is consistent with our results [58].

Iron is a crucial component in the formation of red blood cells [59]. Inadequate nutritional intake of iron contributes to anemia, such as iron deficiency anemia (IDA), which accounts for more than 60% of anemia throughout the world [12]. Our study found a negative association between anemia and the consumption of red meat, corn, and sweet potatoes in women, which is consistent with previous findings [60]. Red meat (particularly pork) is a common source of iron for the Chinese population and is easily absorbed due to the presence of heme iron [61]. This is in conjunction with studies showing a positive relationship between heme iron intake and iron status in the Western population. However, red meat also contains elevated levels of homocysteine, which can increase the risk of cardiovascular diseases, stroke, and colorectal cancer [62, 63]. Interestingly, our study suggested that rarely eating rice (≤ 3 times per month) may be negatively correlated with anemia in women, which is potentially due to the high phytate content that inhibits iron absorption [64]. However, in our study population, nearly all of the participants (98.55%) ate rice 5–7 days per week, with only a small proportion (0.62%) eating rice less frequently. Further investigations are needed to confirm the relationship between rice consumption and anemia. Additionally, our analysis showed that diastolic blood pressure and body fat percentage were negatively correlated with anemia in women.

Compared with women, fewer studies have focused on anemia in males. In this study, we observed that the prevalence of anemia in men increased with age. For men, consuming corn, sweet potatoes, and fruit for 5–7 days per week was negatively correlated with anemia. This effect may be related to the high levels of vitamins C, and B that are found in fruits, corn, or sweet potatoes. Vitamin C increases the bioavailability of nonheme iron, thus leading to an increase in red blood cell content and Hb concentration. The body water percentage index has been proposed for decades, but more research is needed to examine its relationship with anemia. This study found that a body water percentage below normal was negatively related to anemia compared with the a normal body water percentage. Conversely, a body water percentage above normal was positively correlated with anemia in men. Recent studies have demonstrated that increasing plasma volume is due to hemodilution, which can be explained by approximately half of the cases of anemia [65]. A previous study found that a high total body water percentages were associated with a high erythropoietin resistance index, thus decreasing the red blood cell production [66]. In addition, plasma volume excess was associated with anemia [67]. Furthermore, the excessive body water percentage may indicate malnutrition [68, 69]. Thus, the control of body water may be of great significance to male anemia. Our study found an inverse relationship between diastolic blood pressure and anemia in both men and women. These findings warrant further investigation and assessment.

### Strengths and limitations

The strength of our study was that we recruited a relatively large representative sample population of Zhuang from ethnic minority areas, which enabled us to identify the prevalence of anemia and explore risk factors for anemia in such thalassemia high-frequency heterozygous populations. In addition, the study collected data on the basics parameters of diet, physical examination, lifestyle, and sociodemographic variables. These comprehensive exposure variables were selected by using the LASSO regression, which showed that the coefficients of some less contributive variables are forced to be precisely zero. Only the most significant variables are kept in the final model, thus resulting in valid and robust results in the study. The study also had several limitations. First, the Zhuang population accounts for more than 90% of the study; however, the other ethnic groups have small numbers. Thus, the results of this study can only be limited to identifying the anemia prevalence of Zhuang agglomeration areas in Guangxi. Second, including both natural population sampling and physical examination population recruitment strategies may introduce some limitations to the generalizability of our findings. Third, we have not obtained the participants’ genetic information. Therefore, we cannot analyze the effect of the interaction between heredity and the environment on anemia. Fourth, we used a simple food frequency questionnaire to obtain food consumption information, which made it impossible for us to quantitatively analyze the relationship between food consumption and anemia, which should be examined in the future.

## Conclusions

In conclusion, this study was the first to report the use of LASSO and logistic regression for variable selection and identified and deciphered the role of a multitude of factors in anemia by using a population-based design in conjunction with a powerful regularization technique. Our study showed that aging was positively associated with anemia among women and men. In particular, policy-makers should pay attention to preventing anemia in women of reproductive age and in elderly individuanls. Given that the prevalence of anemia is still high in the Guangxi Zhuang minority region of South China, this study provided anemia prevention strategies at the dietary and nondietary factors, which can be applied to the population level. For example, premenopausal women should increase their red meat intake appropriately and decrease the proportion of rice intake. Moreover, men should increase their fruit consumption while controlling the body water percentage within the standard range. Our study will provide reliable evidence for the development of targeted anemia prevention measures in the future.

### Supplementary Information

**Additional file 1:** **Supplemental Table 1.** Sociodemographic and lifestyle factors associated with anemia in univariate analysis in women and men. **Supplemental Table 2.** Anthropometric and body composition factors associated with anemia in univariate analysis in women and men. **Supplemental Table 3.** Dietary factors associated with anemia in univariate analysis in women and men. **Supplemental Table 4.** Chronic diseases statue associated with anemia in univariate analysis in women and men. **Supplemental Table 5.** physical activity and sleep factors associated with anemia in univariate analysis in women and men. **Supplemental Table 6.** Menopausal and birth history associated with anemia in univariate analysis in women. **Supplemental Figure 1.** Flowchart of participant enrollment.

## Data Availability

Data are available from the corresponding author upon reasonable request.

## Abbreviations

DBP: Diastolic blood pressure
GEMCSCD: Guangxi Ethnic Minority Cohort Study of Chronic Diseases
Hb: Hemoglobin
IQR: Interquartile range
LASSO: Least absolute shrinkage and selection operator
SBP: Systolic blood pressure
