# Supplementary material for: Associations of dietary, sociodemographic, and anthropometric factors with anemia among the Zhuang ethnic adults: a cross-sectional study in Guangxi Zhuang Autonomous Region, China
Source: BMC Public Health. 2023 Oct 6;23:1934. doi: 10.1186/s12889-023-16697-2 (PMC10557179; doi:10.1186/s12889-023-16697-2)
Supplement: Supplementary file 1 — Additional file 1: Supplemental Table 1. Sociodemographic and lifestyle factors associated with anemia in univariate analysis in women and men. Supplemental Table 2. Anthropometric and body composition factors associated with anemia in univariate analysis in women and men. Supplemental Table 3. Dietary factors associated with anemia in univariate analysis in women and men. Supplemental Table 4. Chronic diseases statue associated with anemia in univariate analysis in women and men. Supplemental Table 5. physical activity and sleep factors associated with anemia in univariate analysis in women and men. Supplemental Table 6. Menopausal and birth history associated with anemia in univariate analysis in women. Supplemental Figure 1. Flowchart of participant enrollment. [file 12889_2023_16697_MOESM1_ESM.docx]

| Supplemental Table 1. Sociodemographic and lifestyle factors associated with anemia in univariate analysis in women and men | | | | | | |
| --- | --- | --- | --- | --- | --- | --- |
| Characteristic^1^ | Women | | | Men | | |
|  | No-anemia | Anemia | *p*^2^ | No-anemia | Anemia | *p*^2^ |
| Hemoglobin (g/dL) | 13.40 (12.80–14.10) | 11.2 (10.55–11.60) | < 0.0001 | 15.10 (14.30–15.90) | 12.30 (11.60–12.60) | < 0.0001 |
| Age (Year) |  |  | < 0.0001 |  |  | < 0.0001 |
| 24–39 | 453 (8.18) | 90 (7.24) |  | 687 (11.37) | 47 (7.30) |  |
| 40–49 | 1292 (23.34) | 389 (31.30) |  | 2087 (34.54) | 129 (20.03) |  |
| 50–59 | 1729 (31.23) | 264 (21.24) |  | 1657 (27.42) | 150 (23.29) |  |
| 60–69 | 1589 (28.71) | 359 (28.88) |  | 1249 (20.67) | 228 (35.40) |  |
| 70–82 | 473 (8.54) | 141 (11.34) |  | 362 (5.99) | 90 (13.98) |  |
| Total | 5536 | 1243 |  | 6042 | 644 |  |
| Ethnicity |  |  | 0.5501 |  |  | 0.0095 |
| Others | 310 (5.60) | 75 (6.03) |  | 616 (10.20) | 45 (6.99) |  |
| Zhuang | 5226 (94.40) | 1168 (93.97) |  | 5426 (89.80) | 599 (93.01) |  |
| Residence |  |  | 0.0258 |  |  | < 0.0001 |
| Urban | 698 (12.61) | 186 (14.96) |  | 1974 (32.67) | 146 (22.67) |  |
| Rural | 4838 (87.39) | 1057 (85.04) |  | 4068 (67.33) | 498 (77.33) |  |
| Education degree |  |  | 0.0031 |  |  | < 0.0001 |
| Never been to school | 1137 (20.54) | 300 (24.14) |  | 205 (3.39) | 46 (7.14) |  |
| Primary school | 2538 (45.85) | 536 (43.12) |  | 1574 (26.05) | 211 (32.76) |  |
| Junior school | 1254 (22.65) | 254 (20.43) |  | 2232 (36.94) | 224 (34.78) |  |
| High school | 390 (7.04) | 85 (6.84) |  | 1422 (23.54) | 119 (18.48) |  |
| College or above | 217 (3.92) | 68 (5.47) |  | 609 (10.08) | 44 (6.83) |  |
| Occupation |  |  | 0.5696 |  |  | < 0.0001 |
| Farmer | 3198 (57.77) | 729 (58.65) |  | 2472 (40.91) | 338 (52.48) |  |
| Non-farmer | 2338 (42.23) | 514 (41.35) |  | 3570 (59.09) | 306 (47.52) |  |
| Marital status |  |  | 0.1431 |  |  | 0.0079 |
| Married | 4812 (86.92) | 1061 (85.35) |  | 5527 (91.48) | 569 (88.35) |  |
| Unmarried or divorced | 724 (13.07) | 182 (14.64) |  | 515 (8.52) | 75 (11.65) |  |
| Income (thousand RMB/year) |  |  | 0.1715 |  |  | < 0.0001 |
| < 10 | 1171 (21.15) | 289 (23.25) |  | 794 (13.14) | 136 (21.12) |  |
| 10–29.9 | 2057 (37.16) | 439 (35.32) |  | 1724 (28.53) | 222 (34.47) |  |
| 30–59.9 | 1280 (23.12) | 268 (21.56) |  | 1483 (24.54) | 120 (18.63) |  |
| ≥ 60 | 1028 (18.57) | 247 (19.87) |  | 2041 (33.78) | 166 (25.78) |  |
| Number of families living together |  |  | 0.0785 |  |  | 0.2988 |
| Living alone | 174 (3.14) | 44 (3.54) |  | 267 (4.42) | 37 (5.75) |  |
| 2–3 | 1498 (27.06) | 372 (29.93) |  | 2188 (36.21) | 233 (36.18) |  |
| ≥ 4 | 3864 (69.80) | 827 (66.53) |  | 3587 (59.37) | 374 (58.07) |  |
| Smoking |  |  | 0.6010 |  |  | 0.5081 |
| Never | 5525 (99.80) | 1242 (99.92) |  | 2888 (47.80) | 299 (46.43) |  |
| Current or ever | 11 (0.20) | 1 (0.08) |  | 3154 (52.20) | 345 (53.57) |  |
| Alcohol drinking |  |  | 0.3351 |  |  | < 0.0001 |
| Never | 5003 (90.37) | 1122 (90.27) |  | 1844 (30.52) | 267 (41.46) |  |
| < 1 times/week | 496 (8.96) | 117 (9.41) |  | 2592 (42.90) | 242 (37.58) |  |
| ≥ 1 times/week | 37 (0.67) | 4 (0.32) |  | 1606 (26.58) | 135 (20.96) |  |
| Tea drinking |  |  | 0.6975 |  |  | < 0.0001 |
| Never | 3498 (63.19) | 771 (62.03) |  | 2733 (45.23) | 349 (54.19) |  |
| A few times/year | 1525 (27.55) | 357 (28.72) |  | 1851 (30.64) | 180 (27.95) |  |
| ≥ 1 times/week | 513 (9.27) | 115 (9.25) |  | 1458 (24.13) | 115 (17.86) |  |
| ^1^ Continuous variables were described using the median(interquartile range), and categorical variables were expressed as frequency (n) and proportions (%).  ^2^ Wilcox test was used for continuous variables. Chi-square test was used for categorical variables.  Abbreviations: RMB, Renminbi (the currency unit in China). | | | | | | |

| Supplemental Table 2. Anthropometric and body composition factors associated with anemia in univariate analysis in women and men | | | | | | |
| --- | --- | --- | --- | --- | --- | --- |
| Characteristic^1^ | Women | | | Men | | |
|  | No-anemia | Anemia | *P*^2^ | No-anemia | Anemia | *P*^2^ |
| SBP (mmHg) | 126.00 (113.50–140.50) | 124.00 (111.50–138.50) | 0.0039 | 128.00 (118.00–140.50) | 127.50 (116.50–140.00) | 0.0825 |
| DBP (mmHg) | 79.50 (72.50–87.00) | 77.00 (70.00–84.00) | < 0.0001 | 82.25 (75.50–90.00) | 78.00 (71.50–86.50) | < 0.0001 |
| Body fat rate (%) | 29.50 (23.70–34.50) | 27.30 (21.55–32.30) | < 0.0001 | 16.6 (11.9–21.7) | 14.0 (8.4–19.2) | < 0.0001 |
| Lean muscle mass (kg) | 35.80 (33.20–38.50) | 35.70 (33.20–38.40) | 0.3351 | 50.30 (45.90–54.50) | 47.65 (43.70–52.52) | < 0.0001 |
| Bone mass (kg) | 2.20 (1.90–2.40) | 2.20 (1.90–2.40) | 0.6409 | 2.80 (2.60–3.00) | 2.60 (2.40–2.90) | < 0.0001 |
| FEV1 (L) | 1.29 (1.01–1.61) | 1.29 (1.03–1.60) | 0.7876 | 1.87 (1.39–2.38) | 1.65 (1.23–2.16) | < 0.0001 |
| FVC (L) | 1.72 (1.39–2.09) | 1.73 (1.39–2.12) | 0.8526 | 2.41 (1.89–2.95) | 2.16 (1.65–2.78) | < 0.0001 |
| Abdominal obesity^3^ |  |  | < 0.0001 |  |  | < 0.0001 |
| No | 2780 (50.22) | 725 (58.33) |  | 4523 (74.86) | 547 (84.94) |  |
| Yes | 2756 (49.78) | 518 (41.67) |  | 1519 (25.14) | 97 (15.06) |  |
| Waist-hip rate (WHR) | 0.88 (0.83–0.92) | 0.87 (0.83–0.92) | 0.0353 | 0.90 (0.86–0.94) | 0.89 (0.85–0.93) | < 0.0001 |
| BMI (kg/m^2^) |  |  | < 0.0001 |  |  | < 0.0001 |
| Underweight ( < 18.5) | 315 (5.69) | 100 (8.05) |  | 256 (4.24) | 72 (11.18) |  |
| Normal (18.5–23.9) | 2813 (50.81) | 721 (58.00) |  | 2923 (48.38) | 384 (59.63) |  |
| Overweight (24–27.9) | 1838 (33.20) | 350 (28.16) |  | 2225 (36.83) | 146 (22.67) |  |
| Obesity ( ≥ 28) | 570 (10.30) | 72 (5.79) |  | 638 (10.56) | 42 (6.52) |  |
| Body water percentage (%)^4^ |  |  | < 0.0001 |  |  | < 0.0001 |
| Below normal | 358 (6.47) | 51 (4.10) |  | 2071 (34.28) | 118 (18.32) |  |
| Normal | 4871 (87.98) | 1081 (86.97) |  | 3341 (55.30) | 362 (56.21) |  |
| Above normal | 307 (5.55) | 111 (8.93) |  | 630 (10.43) | 164 (25.47) |  |
| Concentration CO of lung |  |  | 0.5433 |  |  | 0.4693 |
| Normal (level: 1–6) | 5317 (96.08) | 1194 (96.06) |  | 3895 (64.46) | 429 (66.61) |  |
| Moderate (level: 7–10) | 176 (3.18) | 43 (3.46) |  | 934 (15.46) | 89 (13.82) |  |
| High (level: 11–30) | 41 (0.74) | 6 (0.48) |  | 1213 (20.08) | 126 (19.57) |  |
| Visceral fat level |  |  | < 0.0001 |  |  | < 0.0001 |
| Normal (level: 1–4) | 1352 (24.42) | 386 (31.06) |  | 1261 (20.87) | 230 (35.71) |  |
| Mild (level: 5–9) | 3743 (67.61) | 804 (64.68) |  | 1578 (26.12) | 154 (23.91) |  |
| Moderate (level: 10–14) | 418 (7.55) | 50 (4.02) |  | 2448 (40.52) | 195 (30.28) |  |
| High (level: ≥ 15) | 23 (0.42) | 3 (0.24) |  | 755 (12.50) | 65 (10.10) |  |
| ^1^ Continuous variables were described using the median(interquartile range), and categorical variables were expressed as frequency (n) and proportions (%).  ^2^ Wilcox test was used for continuous variables. Chi-square test was used for categorical variables.  ^3^ Abdominal obesity was defined as waist circumference (WC) > 90 cm in men and WC > 80 cm in women.  ^4^ The cut-off value of body water percentage (%) for below normal, normal and above normal (women/men) were: < 45/ < 55, 45–60/55–65, > 60/ > 65. | | | | | | |

| Supplemental Table 3. Dietary factors associated with anemia in univariate analysis in women and men | | | | | | |
| --- | --- | --- | --- | --- | --- | --- |
| Consumption^1, 2^ | Women | | | Men | | |
|  | No-anemia | Anemia | *p*^3^ | No-anemia | Anemia | *p*^3^ |
| Skip breakfast |  |  | 0.9286 |  |  | 0.7656 |
| ≤ 3 times/month or never | 4663 (84.23) | 1052 (84.63) |  | 4835 (80.02) | 519 (80.59) |  |
| 1–3 days/week | 178 (3.22) | 38 (3.06) |  | 382 (6.32) | 36 (5.59) |  |
| 4–7 days/week | 695 (12.55) | 153 (12.31) |  | 825 (13.65) | 89 (13.82) |  |
| Night snack |  |  | 0.4755 |  |  | 0.0248 |
| ≤ 3 times/month or never | 5358 (96.78) | 1198 (96.8) |  | 5382 (89.08) | 594 (92.4) |  |
| 1–3 days/week | 101 (1.83) | 22 (1.77) |  | 457 (7.56) | 39 (6.05) |  |
| 4–7 days/week | 77 (1.39) | 23 (1.85) |  | 203 (3.36) | 11 (1.71) |  |
| Rice |  |  | 0.0248 |  |  | 0.5814 |
| ≤ 3 times/month | 42 (0.76) | 2 (0.16) |  | 38 (0.62) | 2 (0.31) |  |
| 1–4 days/week | 41 (0.74) | 5 (0.40) |  | 58 (0.96) | 7 (1.09) |  |
| 5–7 days/week | 5453 (98.51) | 1236(99.44) |  | 5946 (98.42) | 635 (98.60) |  |
| Cooked wheaten food |  |  | 0.4267 |  |  | < 0.0001 |
| ≤ 3 times/month | 3818 (68.96) | 846 (68.06) |  | 3655 (60.49) | 447 (69.41) |  |
| 1–4 days/week | 1286 (23.23) | 308 (24.78) |  | 1738 (28.77) | 151 (23.45) |  |
| 5–7 days/week | 432 (7.80) | 89 (7.16) |  | 649 (10.74) | 46 (7.14) |  |
| Maize/sweet potatoes |  |  | 0.0248 |  |  | < 0.0001 |
| ≤ 3 times/month | 3815 (68.91) | 887 (71.36) |  | 3928 (65.01) | 466 (72.36) |  |
| 1–4 days/week | 1315 (23.75) | 291 (23.41) |  | 1406 (23.27) | 135 (21.96) |  |
| 5–7 days/week | 406 (7.34) | 65 (5.23) |  | 708 (11.72) | 43 (6.68) |  |
| Red meat |  |  | 0.0132 |  |  | 0.0026 |
| ≤ 3 times/month | 530 (9.57) | 139 (11.18) |  | 393 (6.50) | 51 (7.92) |  |
| 1–4 days/week | 1829 (33.04) | 446 (35.88) |  | 1708 (28.27) | 217 (33.69) |  |
| 5–7 days/week | 3177 (57.39) | 658 (52.94) |  | 3941 (65.23) | 376 (58.39) |  |
| Poultry meat |  |  | 0.9464 |  |  | 0.2306 |
| ≤ 3 times/month | 1117 (20.18) | 256 (20.60) |  | 1067 (17.66) | 127 (19.72) |  |
| 1–4 days/week | 2945 (53.20) | 658 (52.94) |  | 3289 (54.44) | 329 (51.09) |  |
| 5–7 days/week | 1474 (26.62) | 329 (26.46) |  | 1686 (27.90) | 188 (29.19) |  |
| Aquatic product |  |  | 0.0146 |  |  | 0.1728 |
| ≤ 3 times/month | 2256 (40.75) | 451 (36.28) |  | 1941 (32.13) | 227 (35.25) |  |
| 1–4 days/week | 2649 (47.85) | 639 (51.41) |  | 3279 (54.27) | 325 (50.47) |  |
| 5–7 days/week | 631 (11.40) | 153 (12.31) |  | 822 (13.60) | 92 (14.29) |  |
| Egg |  |  | 0.0211 |  |  | 0.0383 |
| ≤ 3 times/month | 2453 (44.31) | 498 (40.06) |  | 2306 (38.17) | 279 (43.32) |  |
| 1–4 days/week | 2341 (42.28) | 559 (44.97) |  | 2822 (46.71) | 276 (42.86) |  |
| 5–7 days/week | 742 (13.40) | 186 (14.97) |  | 914 (15.13) | 89 (13.82) |  |
| Fresh vegetables |  |  | 0.3905 |  |  | 0.2139 |
| ≤ 3 times/month | 82 (1.48) | 25 (2.01) |  | 82 (1.36) | 14 (2.17) |  |
| 1–4 days/week | 252 (4.55) | 58 (4.67) |  | 281 (4.65) | 33 (5.12) |  |
| 5–7 days/week | 5202 (93.97) | 1160 (93.32) |  | 5679 (93.99) | 597 (92.70) |  |
| Picked vegetables |  |  | 0.3403 |  |  | 0.5614 |
| ≤ 3 times/month | 4685 (84.63) | 1034 (83.19) |  | 5099 (84.39) | 553 (85.87) |  |
| 1–4 days/week | 675 (12.19) | 161 (12.95) |  | 804 (13.31) | 76 (11.80) |  |
| 5–7 days/week | 176 (3.18) | 48 (3.86) |  | 139 (2.30) | 15 (2.33) |  |
| Fresh fruit |  |  | 0.9360 |  |  | < 0.0001 |
| ≤ 3 times/month | 1081 (19.53) | 245 (19.71) |  | 1169 (19.35) | 162 (25.16) |  |
| 1–4 days/week | 1981 (35.78) | 438 (35.24) |  | 2091 (34.61) | 248 (38.50) |  |
| 5–7 days/week | 2474 (44.39) | 560 (45.05) |  | 2782 (46.04) | 234 (36.34) |  |
| Nut |  |  | 0.7056 |  |  | 0.005 |
| ≤ 3 times/month | 4505 (81.38) | 999 (80.37) |  | 4529 (74.96) | 518 (80.44) |  |
| 1–4 days/week | 841 (15.19) | 198 (15.93) |  | 1242 (20.56) | 98 (15.22) |  |
| 5–7 days/week | 190 (3.43) | 46 (3.70) |  | 271 (4.49) | 28 (4.34) |  |
| Milk |  |  | 0.7321 |  |  | 0.0472 |
| ≤ 3 times/month | 4845 (87.52) | 1081 (86.97) |  | 5035 (83.33) | 561 (87.11) |  |
| 1–4 days/week | 444 (8.02) | 108 (8.69) |  | 733 (12.13) | 61 (9.47) |  |
| 5–7 days/week | 247 (4.46) | 54 (4.34) |  | 274 (4.54) | 22 (3.42) |  |
| Dessert |  |  | 0.8613 |  |  | 0.8199 |
| ≤ 3 times/month | 4791 (86.54) | 1069 (86.00) |  | 5360 (88.71) | 574 (89.13) |  |
| 1–4 days/week | 642 (11.60) | 151 (12.15) |  | 591 (9.78) | 59 (9.16) |  |
| 5–7 days/week | 103 (1.86) | 23 (1.85) |  | 91 (1.51) | 11 (1.71) |  |
| Soup |  |  | 0.8706 |  |  | 0.0195 |
| ≤ 3 times/month | 1795 (32.42) | 412 (33.15) |  | 2166 (35.85) | 247 (38.35) |  |
| 1–4 days/week | 1927 (34.80) | 425 (34.19) |  | 2037 (33.72) | 182 (28.26) |  |
| 5–7 days/week | 1814 (32.76) | 406 (32.67) |  | 1839 (30.44) | 215 (33.39) |  |
| Vitamin products |  |  | 0.2658 |  |  | 0.8882 |
| No | 5435 (98.8) | 1226 (98.63) |  | 5989 (99.12) | 638 (99.07) |  |
| Yes | 101 (1.82) | 17 (1.37) |  | 53 (0.88) | 6 (0.93) |  |
| Cod liver oil |  |  | 0.4184 |  |  | 1 |
| No | 5514 (99.60) | 1236 (99.44) |  | 6009 (99.45) | 640 (99.38) |  |
| Yes | 22 (0.40) | 7 (0.56) |  | 33 (0.55) | 4 (0.62) |  |
| Calcium, iron and zinc |  |  | 0.3675 |  |  | 0.0133 |
| No | 5121 (92.50) | 1159 (93.24) |  | 5823 (96.38) | 608 (94.41) |  |
| Yes | 415 (7.50) | 84 (6.67) |  | 219 (3.62) | 36 (5.59) |  |
| Yogurt |  |  | 0.6621 |  |  | 0.0240 |
| No | 5321 (96.12) | 1198 (96.37) |  | 5767 (95.45) | 627 (97.36) |  |
| Yes | 215 (3.89) | 45 (3.62) |  | 275 (4.55) | 17 (2.64) |  |
| ^1^ The frequency of traditional food consumption in the past year was obtained.  ^2^ Categorical variables were expressed as frequency (n) and proportions (%).  ^3^ Chi-square test was used. | | | | | | |

| Supplemental Table 4. Chronic diseases statue associated with anemia in univariate analysis in women and men | | | | | | |
| --- | --- | --- | --- | --- | --- | --- |
| Diseases^1^ | Women | | | Men | | |
|  | No-anemia | Anemia | *p*^2^ | No-anemia | Anemia | *p*^2^ |
| Hypertension^3^ |  |  | 0.0043 |  |  | 0.8049 |
| No | 4678 (84.50) | 1090 (87.69) |  | 5157 (85.35) | 552 (85.71) |  |
| Yes | 858 (15.50) | 153 (12.31) |  | 885 (14.65) | 92 (14.29) |  |
| Diabetes^4^ |  |  | 0.2490 |  |  | 0.0114 |
| No | 5349 (96.62) | 1209 (97.26) |  | 5862 (97.02) | 613 (95.19) |  |
| Yes | 187 (3.38) | 34 (2.74) |  | 180 (2.98) | 31 (4.81) |  |
| CHD^5^ |  |  | 0.8963 |  |  | 0.1716 |
| No | 5476 (98.92) | 1229 (98.87) |  | 5968 (98.78) | 632 (98.14) |  |
| Yes | 60 (1.18) | 14 (1.13) |  | 74 (1.22) | 12 (1.86) |  |
| Stroke^6^ |  |  | 0.8680 |  |  | 0.0054 |
| No | 5498 (99.31) | 1235 (99.36) |  | 5983 (99.02) | 630 (97.83) |  |
| Yes | 38 (0.69) | 8 (0.64) |  | 59 (0.98) | 14 (2.17) |  |
| Cancer^7^ |  |  | 1 |  |  | 0.0009 |
| No | 5521 (99.73) | 1240 (99.76) |  | 6028 (99.77) | 637 (98.91) |  |
| Yes | 15 (0.27) | 3 (0.24) |  | 14 (0.23) | 7 (1.07) |  |
| Family history of chronic diseases^8^ |  |  | 0.2193 |  |  | 0.0015 |
| No | 830 (14.99) | 166 (13.36) |  | 1337 (22.13) | 120 (18.63) |  |
| Yes | 4184 (75.58) | 968 (77.88) |  | 4279 (70.82) | 456 (70.81) |  |
| Unknown | 522 (9.43) | 109 (8.77) |  | 426 (7.05) | 68 (10.56) |  |
| Antibiotic history^9^ |  |  | 0.1112 |  |  | 0.0152 |
| No | 5259 (95.00) | 1167 (93.89) |  | 5572 (92.22) | 611 (94.88) |  |
| Yes | 277 (5.00) | 76 (6.11) |  | 470 (7.78) | 33 (5.12) |  |
| ^1^ Categorical variables were expressed as frequency (n) and proportions (%).  ^2^ Chi-square test was used.  ^3^ Hypertension was defined as self-reported physician diagnosis of hypertension.  ^4^ Diabetes was defined as self-reported physician diagnosis of diabates.  ^5^ CHD: Coronary Heart Diseases, was defined as self-reported physician diagnosis of CHD.  ^6^ Stroke was defined as self-reported physician diagnosis of ischemic or hemorrhagic strokes.  ^7^ Cancer was defined as self-reported physician diagnosis of any primary malignant tumor.  ^8^ Family history of chronic diseases refers to participants who have at least one first-degree relative (parent, sibling, or child) who has been diagnosed with any of the following conditions: hypertension, diabetes, coronary heart disease (CHD), stroke, or cancer.  ^9^ Whether the participants have taken antibiotics in the past three months. | | | | | | |

| Supplemental Table 5. physical activity and sleep factors associated with anemia in univariate analysis in women and men | | | | | | |
| --- | --- | --- | --- | --- | --- | --- |
| Characteristic^1^ | Women | | | Men | | |
|  | No-anemia | Anemia | *p*^2^ | No-anemia | Anemia | *p*^2^ |
| Intense exercise |  |  | 0.2564 |  |  | 0.2432 |
| No | 4348 (78.54) | 958 (77.07) |  | 4390 (72.66) | 454 (70.50) |  |
| Yes | 1188 (21.46) | 285 (22.93) |  | 1652 (27.34) | 190 (29.50) |  |
| Moderate exercise |  |  | 0.0457 |  |  | 0.2074 |
| No | 3603 (65.08) | 846 (68.06) |  | 4170 (69.02) | 460 (71.43) |  |
| Yes | 1933 (34.92) | 397 (31.94) |  | 1872 (31.98) | 184 (28.57) |  |
| Walking ( ≥ 10 minutes/day) |  |  | 0.4976 |  |  | 0.0414 |
| No | 1449 (26.17) | 337 (27.11) |  | 1594 (26.38) | 194 (30.12) |  |
| Yes | 4087 (73.83) | 906 (72.89) |  | 4448 (73.62) | 450 (69.88) |  |
| Sitting |  |  | 0.8691 |  |  | 0.2067 |
| < 6 hours/day | 4979 (89.94) | 1116 (89.78) |  | 5261 (87.07) | 572 (88.82) |  |
| ≥ 6 hours/day | 557 (10.06) | 127 (10.22) |  | 781 (12.93) | 72 (11.18) |  |
| Time of going bed |  |  | 0.4086 |  |  | < 0.0001 |
| Before 22:00 | 1379 (24.91) | 287 (23.09) |  | 959 (15.87) | 141 (21.89) |  |
| 22:00–23:00 | 2281 (41.20) | 514 (41.35) |  | 1739 (28.78) | 207 (32.14) |  |
| 23:00–24:00 | 1330 (24.02) | 322 (25.91) |  | 2003 (33.15) | 184 (28.57) |  |
| After 00:00 | 546 (9.86) | 120 (9.75) |  | 1341 (22.20) | 112 (17.39) |  |
| Total sleeping time |  |  | 0.6391 |  |  | 0.1545 |
| < 7 hours per night | 1393 (25.16) | 313 (25.18) |  | 1643 (27.19) | 159 (24.69) |  |
| 7–7.9 hours per night | 1680 (30.35) | 385 (30.97) |  | 2042 (33.80) | 210 (32.6) |  |
| 8–8.9 hours per night | 1715 (30.98) | 365 (29.36) |  | 1637 (27.09) | 181 (28.1) |  |
| ≥ 9 hours per night | 748 (13.51) | 180 (14.48) |  | 720 (11.92) | 94 (14.6) |  |
| PSQI score |  |  | 0.2632 |  |  | 0.3704 |
| ≤ 5 | 3501 (63.24) | 765 (61.54) |  | 4397 (72.77) | 458 (71.12) |  |
| > 5 | 2035 (36.76) | 478 (38.56) |  | 1645 (27.23) | 186 (28.88) |  |
| Afternoon nap |  |  | 0.8840 |  |  | 0.2419 |
| No | 2435 (43.98) | 553 (44.49) |  | 2346 (38.83) | 269 (41.77) |  |
| Only in summer | 467 (8.44) | 100 (8.05) |  | 362 (5.99) | 42 (6.52) |  |
| All year round | 2634 (47.58) | 590 (47.47) |  | 3334 (55.18) | 333 (51.71) |  |
| ^1^ Categorical variables were expressed as frequency (n) and proportions (%). ^2^ Chi-square test was used. | | | | | | |

| Supplemental Table 6. Menopausal and birth history associated with anemia in univariate analysis in women | | | |
| --- | --- | --- | --- |
| Characteristic^1^ | No-anemia | Anemia | *p*^2^ |
| Menopause |  |  | < 0.0001 |
| No | 2004 (36.19) | 536 (43.12) |  |
| Yes | 3532 (63.80) | 707 (56.88) |  |
| Menarche age (year) | 15 (14–16) | 15 (14–16) | 0.8329 |
| Number of pregnancies |  |  | 0.9316 |
| 0 | 103 (1.86) | 25 (2.01) |  |
| 1–3 | 2817 (50.88) | 629 (50.60) |  |
| ≥ 4 | 2616 (47.25) | 589 (47.39) |  |
| Number of live birth |  |  | 0.6563 |
| 0 | 190 (3.43) | 47 (3.78) |  |
| 1–3 | 3671 (66.31) | 809 (65.08) |  |
| ≥ 4 | 1675 (30.26) | 387 (31.13) |  |
| Age of first birth(year) |  |  | 0.3337 |
| None | 141 (2.55) | 39 (3.14) |  |
| 14–24 | 3270 (59.07) | 704 (56.64) |  |
| 25–35 | 2068 (37.36) | 485 (39.02) |  |
| ≥ 36 | 57 (1.03) | 15 (1.21) |  |
| ^1^ Continuous variables were described using the median (interquartile range), and categorical variables were expressed as frequency (n) and proportions (%).  ^2^ Wilcox test was used for continuous variables. Chi-square test was used for categorical variables. | | | |


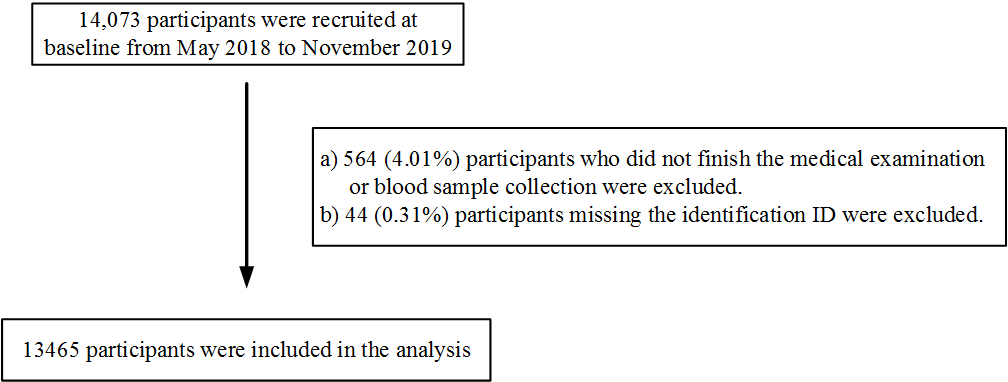


**Supplemental Figure 1** Flowchart of participant enrollment.
